# 2,2′-[(2,2′-Bipyridine-3,3′-di­yl)bis­(nitrilo­methyl­idyne)]diphenol

**DOI:** 10.1107/S1600536809032346

**Published:** 2009-08-29

**Authors:** Hong Liang Li

**Affiliations:** a Department of Chemistry, Dezhou University, Dezhou 253023, People’s Republic of China

## Abstract

The title mol­ecule, C_24_H_18_N_4_O_2_, lies on a twofold rotation axis with a dihedral angle of 73.7 (1)° between the mean planes of the symmetry-related pyridine rings. The dihedral angle between unique benzene and pyridine rings is 8.0 (1)°. An intra­molecular O—H⋯N hydrogen bond may influence the mol­ecular conformation. In the crystal structure, there are weak π–π stacking inter­actions with a centroid–centroid distance of 3.7838 (15) Å.

## Related literature

For background to the use of 2,2-bipyridine derivatives in coordination chemistry, see: Stephenson & Hardie (2007 ▶); Hou *et al.* (2008*a* ▶,*b* ▶). For a related structure, see: Rice *et al.* (2002 ▶).
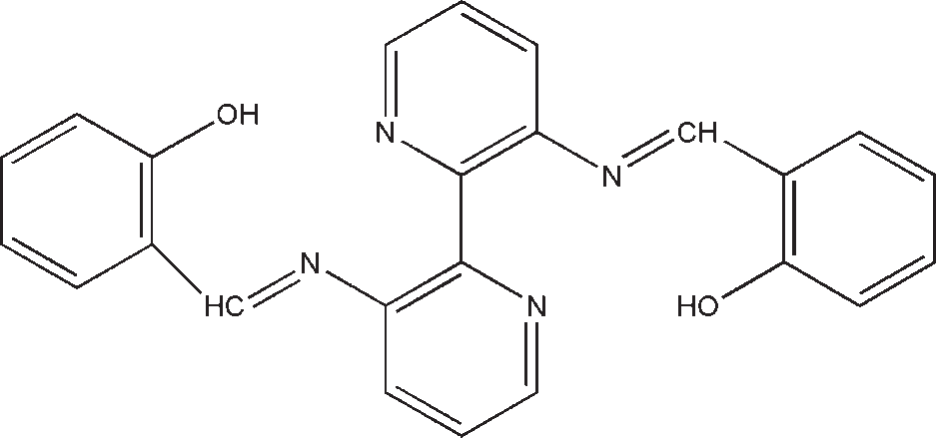


## Experimental

### 

#### Crystal data

C_24_H_18_N_4_O_2_
*M* *_r_* = 394.42
Monoclinic, 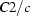

*a* = 21.017 (3) Å
*b* = 8.4485 (14) Å
*c* = 13.012 (2) Å
β = 121.980 (3)°
*V* = 1959.8 (5) Å^3^
*Z* = 4
Mo *K*α radiation
μ = 0.09 mm^−1^
*T* = 298 K
0.38 × 0.20 × 0.16 mm

#### Data collection

Bruker SMART CCD diffractometer
Absorption correction: multi-scan (*SADABS*; Sheldrick, 1996 ▶) *T*_min_ = 0.967, *T*_max_ = 0.986
5242 measured reflections
1913 independent reflections
1334 reflections with *I* > 2σ(*I*)
*R*_int_ = 0.025

#### Refinement

*R*[*F* ^2^ > 2σ(*F* ^2^)] = 0.055
*wR*(*F* ^2^) = 0.143
*S* = 1.02
1913 reflections
137 parameters
H-atom parameters constrained
Δρ_max_ = 0.17 e Å^−3^
Δρ_min_ = −0.13 e Å^−3^

### 

Data collection: *SMART* (Bruker, 1997 ▶); cell refinement: *SAINT* (Bruker, 1997 ▶); data reduction: *SAINT*; program(s) used to solve structure: *SHELXTL* (Sheldrick, 2008 ▶); program(s) used to refine structure: *SHELXTL*; molecular graphics: *SHELXTL*; software used to prepare material for publication: *SHELXTL*.

## Supplementary Material

Crystal structure: contains datablocks I, global. DOI: 10.1107/S1600536809032346/lh2878sup1.cif

Structure factors: contains datablocks I. DOI: 10.1107/S1600536809032346/lh2878Isup2.hkl

Additional supplementary materials:  crystallographic information; 3D view; checkCIF report

## Figures and Tables

**Table 1:** Hydrogen-bond geometry (Å, °)

*D*—H⋯*A*	*D*—H	H⋯*A*	*D*⋯*A*	*D*—H⋯*A*
O1—H1⋯N1	0.82	1.89	2.619 (2)	147

**Fig. 1.**
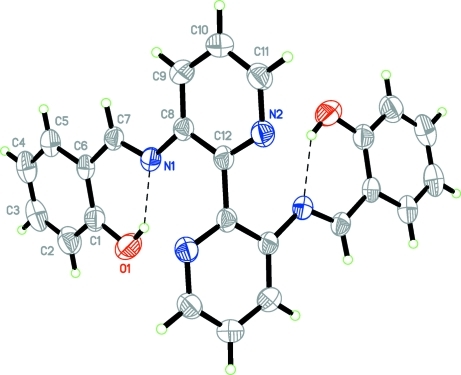
The molecular structure of the title compound with displacement ellipsoids drawn at the 30% probability level. The dashed lines indicate hydrogen bonds and the unlabeled atoms are related by the symmetry code (-x, y, -z+1/2).

## supplementary crystallographic information

### Comment

Derivatives of 2,2-bipyridine are useful ligands and  play an important role in modern coordination chemistry (Stephenson & Hardie, 2007; Hou *et al.*, 2008*a*,*b*). The title compound (I) was synthesized as a potential ligand and its crystal structure is reported herein. The molecular structure of (I) is shown in Fig. 1. The molecule lies on a twofold rotation axis with dihedral angle of 73.7 (1)° between the mean planes of the symmetry related pyridine rings. In the related crystal structure of 3,3'-diamino-2,2'-dipyridine which has already been reported (Rice *et al.* 2002) the molecule lies on an inversion center and the mean planes of the two pyridine are essentially co-planar. The large deviation from planarity in (I) can be expected in terms of steric relief with respect to the bulky 2-(methylimino)phenol substituents. An intramolecular O—H···N hydrogen bond may influence the molecular conformation. In the crystal structure, there is a weak π–π stacking interactions involving pyridine rings and symmetry-related benzene rings with the relevant geometry being *Cg*1···*Cg*2^i^ = 3.7838 (15) Å, *Cg*1···*Cg*2^i^_perp_ = 3.353 Å and α = 7.97° [symmetry code: (i) -*x*, 1-*y*, -*z*; *Cg*1 and *Cg*2 are the centroids of the C8—C12/N2 ring and C1—C6 ring, respectively; *Cg*1···*Cg*2^1^_perp_ is the perpendicular distance from ring *Cg*1 to ring *Cg*2^i^; α is the dihedral between ring plane *Cg*1 and ring plane *Cg*2^i^].

### Experimental

A 35 ml methanol solution of 3,3'-diamino-2,2'-dipyridine (1.03 g, 5.53 mmol) was added to salicylaldehyde (1.40 g, 11.46 mmol) and the mixture was stirred and refluxed for 5 h. Yellow single crystals were obtained after the filtrate had been allowed to stand at room temperature for three weeks.

### Refinement

All H atoms were placed in in calculated positions and refined as riding with C—H = 0.93 Å and *U*_iso_(H) = 1.2*U*_eq_(C), O—H = 0.82 Å and *U*_iso_(H) = 1.5*U*_eq_(O).

### Crystal data

**Table:**

C_24_H_18_N_4_O_2_	*F*(000) = 824
*M**_r_* = 394.42	*D*_x_ = 1.337 Mg m^−^^3^
Monoclinic, *C*2/*c*	Mo *K*α radiation, λ = 0.71073 Å
Hall symbol: -C 2yc	Cell parameters from 1047 reflections
*a* = 21.017 (3) Å	θ = 2.7–22.4°
*b* = 8.4485 (14) Å	µ = 0.09 mm^−^^1^
*c* = 13.012 (2) Å	*T* = 298 K
β = 121.980 (3)°	Block, yellow
*V* = 1959.8 (5) Å^3^	0.38 × 0.20 × 0.16 mm
*Z* = 4	

### Data collection

**Table:**

Bruker SMART CCD diffractometer	1913 independent reflections
Radiation source: fine-focus sealed tube	1334 reflections with *I* > 2σ(*I*)
graphite	*R*_int_ = 0.025
φ and ω scans	θ_max_ = 26.0°, θ_min_ = 2.3°
Absorption correction: multi-scan (*SADABS*; Sheldrick, 1996)	*h* = −21→25
*T*_min_ = 0.967, *T*_max_ = 0.986	*k* = −9→10
5242 measured reflections	*l* = −15→16

### Refinement

**Table:**

Refinement on *F*^2^	Primary atom site location: structure-invariant direct methods
Least-squares matrix: full	Secondary atom site location: difference Fourier map
*R*[*F*^2^ > 2σ(*F*^2^)] = 0.055	Hydrogen site location: inferred from neighbouring sites
*wR*(*F*^2^) = 0.143	H-atom parameters constrained
*S* = 1.02	*w* = 1/[σ^2^(*F*_o_^2^) + (0.063*P*)^2^ + 0.6383*P*] where *P* = (*F*_o_^2^ + 2*F*_c_^2^)/3
1913 reflections	(Δ/σ)_max_ < 0.001
137 parameters	Δρ_max_ = 0.17 e Å^−^^3^
0 restraints	Δρ_min_ = −0.13 e Å^−^^3^

### Special details

**Table:**

Geometry. All e.s.d.'s (except the e.s.d. in the dihedral angle between two l.s. planes) are estimated using the full covariance matrix. The cell e.s.d.'s are taken into account individually in the estimation of e.s.d.'s in distances, angles and torsion angles; correlations between e.s.d.'s in cell parameters are only used when they are defined by crystal symmetry. An approximate (isotropic) treatment of cell e.s.d.'s is used for estimating e.s.d.'s involving l.s. planes.
Refinement. Refinement of *F*^2^ against ALL reflections. The weighted *R*-factor *wR* and goodness of fit *S* are based on *F*^2^, conventional *R*-factors *R* are based on *F*, with *F* set to zero for negative *F*^2^. The threshold expression of *F*^2^ > σ(*F*^2^) is used only for calculating *R*-factors(gt) *etc*. and is not relevant to the choice of reflections for refinement. *R*-factors based on *F*^2^ are statistically about twice as large as those based on *F*, and *R*- factors based on ALL data will be even larger.

### Fractional atomic coordinates and isotropic or equivalent isotropic displacement parameters (Å^2^)

**Table:**

	*x*	*y*	*z*	*U*_iso_*/*U*_eq_	
C1	0.12783 (13)	0.8486 (3)	0.1341 (2)	0.0702 (6)	
C2	0.18125 (15)	0.9514 (3)	0.1412 (3)	0.0917 (8)	
H2	0.2263	0.9648	0.2147	0.110*	
C3	0.16874 (18)	1.0339 (3)	0.0413 (3)	0.0928 (9)	
H3	0.2052	1.1029	0.0480	0.111*	
C4	0.10274 (19)	1.0156 (3)	−0.0687 (3)	0.0867 (8)	
H4	0.0946	1.0706	−0.1366	0.104*	
C5	0.04893 (14)	0.9146 (3)	−0.0766 (2)	0.0709 (6)	
H5	0.0042	0.9023	−0.1507	0.085*	
C6	0.05958 (12)	0.8302 (2)	0.02338 (19)	0.0567 (5)	
C7	0.00038 (11)	0.7307 (2)	0.01146 (18)	0.0553 (5)	
H7	−0.0435	0.7208	−0.0642	0.066*	
C8	−0.05332 (10)	0.5646 (2)	0.09104 (17)	0.0513 (5)	
C9	−0.12535 (12)	0.5543 (3)	−0.01036 (19)	0.0666 (6)	
H9	−0.1379	0.6077	−0.0812	0.080*	
C10	−0.17782 (13)	0.4647 (3)	−0.0050 (2)	0.0723 (6)	
H10	−0.2263	0.4563	−0.0722	0.087*	
C11	−0.15804 (12)	0.3878 (3)	0.1000 (2)	0.0681 (6)	
H11	−0.1942	0.3269	0.1019	0.082*	
C12	−0.03848 (11)	0.4816 (2)	0.19397 (17)	0.0516 (5)	
N1	0.00610 (9)	0.65566 (19)	0.10122 (14)	0.0549 (4)	
N2	−0.08982 (9)	0.3951 (2)	0.20004 (15)	0.0621 (5)	
O1	0.14272 (9)	0.7671 (3)	0.23344 (14)	0.0978 (6)	
H1	0.1053	0.7176	0.2190	0.147*	

### Atomic displacement parameters (Å^2^)

**Table:**

	*U*^11^	*U*^22^	*U*^33^	*U*^12^	*U*^13^	*U*^23^
C1	0.0733 (15)	0.0801 (15)	0.0708 (16)	−0.0074 (12)	0.0475 (13)	−0.0130 (12)
C2	0.0844 (19)	0.105 (2)	0.102 (2)	−0.0209 (15)	0.0600 (17)	−0.0259 (17)
C3	0.107 (2)	0.0738 (17)	0.142 (3)	−0.0137 (15)	0.097 (2)	−0.0202 (18)
C4	0.121 (2)	0.0668 (16)	0.119 (2)	0.0108 (15)	0.096 (2)	0.0133 (15)
C5	0.0889 (16)	0.0662 (14)	0.0815 (16)	0.0107 (12)	0.0614 (14)	0.0075 (12)
C6	0.0717 (14)	0.0539 (12)	0.0624 (13)	0.0046 (9)	0.0477 (12)	−0.0032 (10)
C7	0.0638 (12)	0.0581 (12)	0.0522 (12)	0.0057 (9)	0.0363 (10)	−0.0029 (9)
C8	0.0561 (12)	0.0530 (11)	0.0510 (12)	0.0017 (9)	0.0327 (10)	−0.0056 (9)
C9	0.0669 (14)	0.0753 (15)	0.0553 (13)	0.0011 (11)	0.0308 (11)	0.0047 (11)
C10	0.0587 (13)	0.0854 (16)	0.0628 (14)	−0.0059 (11)	0.0255 (11)	−0.0063 (12)
C11	0.0602 (13)	0.0752 (15)	0.0751 (15)	−0.0104 (11)	0.0401 (12)	−0.0098 (12)
C12	0.0569 (12)	0.0538 (11)	0.0544 (12)	0.0000 (9)	0.0364 (9)	−0.0069 (9)
N1	0.0613 (10)	0.0588 (10)	0.0524 (10)	0.0019 (8)	0.0353 (8)	0.0009 (8)
N2	0.0619 (11)	0.0686 (11)	0.0634 (11)	−0.0071 (9)	0.0385 (9)	−0.0041 (9)
O1	0.0857 (13)	0.1405 (17)	0.0616 (11)	−0.0203 (11)	0.0352 (9)	0.0015 (10)

### Geometric parameters (Å, °)

**Table:**

C1—O1	1.347 (3)	C7—H7	0.9300
C1—C2	1.383 (3)	C8—C9	1.387 (3)
C1—C6	1.403 (3)	C8—C12	1.393 (3)
C2—C3	1.372 (4)	C8—N1	1.412 (2)
C2—H2	0.9300	C9—C10	1.369 (3)
C3—C4	1.376 (4)	C9—H9	0.9300
C3—H3	0.9300	C10—C11	1.364 (3)
C4—C5	1.377 (3)	C10—H10	0.9300
C4—H4	0.9300	C11—N2	1.333 (3)
C5—C6	1.393 (3)	C11—H11	0.9300
C5—H5	0.9300	C12—N2	1.340 (2)
C6—C7	1.440 (3)	C12—C12^i^	1.498 (4)
C7—N1	1.278 (2)	O1—H1	0.8200
			
O1—C1—C2	119.3 (2)	C6—C7—H7	118.9
O1—C1—C6	121.3 (2)	C9—C8—C12	117.31 (18)
C2—C1—C6	119.4 (2)	C9—C8—N1	126.07 (18)
C3—C2—C1	120.9 (3)	C12—C8—N1	116.59 (17)
C3—C2—H2	119.5	C10—C9—C8	119.3 (2)
C1—C2—H2	119.5	C10—C9—H9	120.4
C2—C3—C4	120.7 (3)	C8—C9—H9	120.4
C2—C3—H3	119.7	C11—C10—C9	119.2 (2)
C4—C3—H3	119.7	C11—C10—H10	120.4
C3—C4—C5	118.9 (3)	C9—C10—H10	120.4
C3—C4—H4	120.6	N2—C11—C10	123.8 (2)
C5—C4—H4	120.6	N2—C11—H11	118.1
C4—C5—C6	121.9 (2)	C10—C11—H11	118.1
C4—C5—H5	119.1	N2—C12—C8	123.65 (18)
C6—C5—H5	119.1	N2—C12—C12^i^	115.52 (18)
C5—C6—C1	118.2 (2)	C8—C12—C12^i^	120.82 (18)
C5—C6—C7	119.7 (2)	C7—N1—C8	122.31 (17)
C1—C6—C7	122.05 (19)	C11—N2—C12	116.79 (18)
N1—C7—C6	122.25 (19)	C1—O1—H1	109.5
N1—C7—H7	118.9		
			
O1—C1—C2—C3	178.6 (2)	N1—C8—C9—C10	−177.84 (18)
C6—C1—C2—C3	−0.8 (4)	C8—C9—C10—C11	0.1 (3)
C1—C2—C3—C4	−0.3 (4)	C9—C10—C11—N2	0.5 (3)
C2—C3—C4—C5	0.8 (4)	C9—C8—C12—N2	−0.7 (3)
C3—C4—C5—C6	−0.2 (3)	N1—C8—C12—N2	177.33 (16)
C4—C5—C6—C1	−0.9 (3)	C9—C8—C12—C12^i^	177.85 (16)
C4—C5—C6—C7	177.60 (19)	N1—C8—C12—C12^i^	−4.1 (2)
O1—C1—C6—C5	−178.0 (2)	C6—C7—N1—C8	176.66 (16)
C2—C1—C6—C5	1.4 (3)	C9—C8—N1—C7	−5.5 (3)
O1—C1—C6—C7	3.5 (3)	C12—C8—N1—C7	176.58 (17)
C2—C1—C6—C7	−177.06 (19)	C10—C11—N2—C12	−1.1 (3)
C5—C6—C7—N1	−177.25 (18)	C8—C12—N2—C11	1.3 (3)
C1—C6—C7—N1	1.2 (3)	C12^i^—C12—N2—C11	−177.40 (16)
C12—C8—C9—C10	0.0 (3)		

Symmetry codes: (i) −*x*, *y*, −*z*+1/2.

### Hydrogen-bond geometry (Å, °)

**Table:**

*D*—H···*A*	*D*—H	H···*A*	*D*···*A*	*D*—H···*A*
O1—H1···N1	0.82	1.89	2.619 (2)	147
